# Novel Applications of Natural Biomaterials in Dentistry—Properties, Uses, and Development Perspectives

**DOI:** 10.3390/ma18092124

**Published:** 2025-05-05

**Authors:** Magdalena Paczkowska-Walendowska, Maciej Kulawik, Jakub Kwiatek, Dimitrios Bikiaris, Judyta Cielecka-Piontek

**Affiliations:** 1 Department of Pharmacognosy and Biomaterials, Poznan University of Medical Sciences, 61-701 Poznan, Poland; maciej.kulawik@student.ump.edu.pl (M.K.); jpiontek@ump.edu.pl (J.C.-P.); 2 Kwiatek Dental Clinic Sp. Z.o.o., Kordeckiego 22, 60-144 Poznan, Poland; jakubkwiatek@klinikakwiatek.pl; 3 Laboratory of Polymer Chemistry and Technology, Department of Chemistry, Aristotle University of Thessaloniki, 541 24 Thessaloniki, Greece; dbic@chem.auth.gr

**Keywords:** natural biomaterials, stomatology, periodontal disease, caries, implant coating, bone grafts

## Abstract

Natural biomaterials have gained significant attention in modern dentistry due to their biocompatibility, biodegradability, and low immunogenicity. These materials, including alginate, cellulose, chitosan, collagen, and hydroxyapatite, have been widely explored for their applications in stomatology. They play a crucial role in periodontal disease treatment, caries prevention, and implantology, providing an alternative to synthetic materials. Natural polymers such as chitosan and cellulose are utilized in drug delivery systems and tissue regeneration, while hydroxyapatite serves as a bone substitute due to its osteoconductive properties. Collagen-based scaffolds and coatings enhance periodontal and bone tissue regeneration. Additionally, bioengineered and chemically modified biomaterials offer improved mechanical and biological characteristics, expanding their clinical applications. This review aims to provide a comprehensive analysis of the biological properties, advantages, and limitations of selected natural biomaterials in dentistry. It explores their applications in various aspects of stomatology, including periodontal disease prevention and regeneration, dental caries prevention, bone substitutes in implantology, and dental implant coating. Although natural biomaterials exhibit promising properties, further research is necessary to refine their performance, enhance stability, and ensure long-term safety. Advancements in nanotechnology and bioengineering continue to drive the development of innovative natural biomaterials, paving the way for more effective and biocompatible dental therapies.

## 1. Introduction

The history of biomaterials and their use in dentistry dates back thousands of years. However, the rapid development of stomatology began only in the last three centuries [1]. Nearly half of the people on Earth suffer from pain and discomfort caused by oral diseases. The health of the teeth and the tissues surrounding them is important for maintaining overall health and well-being [2]. As is known, diseases such as periodontal disease, tooth decay, and implant-related problems are multifactorial. Because the oral cavity is the first part of the digestive system and is exposed to the external environment, its microbiome dynamics are highly active. Due to the complexity and prevalence of oral diseases, extensive measures are being taken to restore normal oral function [3]. To meet dental challenges, natural biomaterials have emerged as a promising solution in modern dentistry. Compared to artificial materials, they perform more favorably in selected applications [4].

The growing interest in naturally derived biomaterials is also due to their sourcing methods. They are often produced from renewable sources, which makes them attractive. In addition, advances in their bioengineering, chemical modifications, and the development of nanotechnology have further increased their potential and mitigated some of their drawbacks. This article aims to thoroughly analyze the biological properties of selected natural biomaterials. In addition, this paper focuses on the applications of the described materials in selected aspects of dentistry.

## 2. Materials and Methods

The databases Google Scholar, PubMed, Scopus, and Web of Science were searched for studies on the therapeutic efficacy of natural biomaterials, in terms of dental applications. Keywords included biopolymers, natural biopolymers, alginate, cellulose, chitosan, collagen, cyclodextrins, gelatin, hyaluronic acid, hydroxyapatite, silk, dental, wound healing, periodontitis, dental caries, dental implants, and combinations. The following inclusion criteria were adopted: original English-language articles published in peer-reviewed journals between 2005 and 2024, while, for application papers, between 2020 and 2024.

This review is limited by its focus on English-language publications and selected databases, with a specific dental indication. Variability in biomaterial sourcing and processing also complicates comparisons between studies. Many applications discussed are still in early clinical stages, requiring further large-scale validation. Additionally, no meta-analysis was performed to statistically assess outcomes.

## 3. Characteristics of Selected Natural Biomaterials Used in Dentistry

Biomaterials are materials that can interact with biological organisms without causing adverse effects. For a biomaterial to be used in regenerative medicine, it must possess several key characteristics, such as non-toxicity and an appropriate chemical composition. The surface topography of a biomaterial plays a crucial role in its interaction with tissues [5,6]. Natural biomaterials are particularly attractive due to their favorable properties, including low immunogenicity, wide availability, and affordability. Additionally, they are biodegradable and well tolerated by the body [6,7].

### 3.1. Alginate

**Figure 1:**
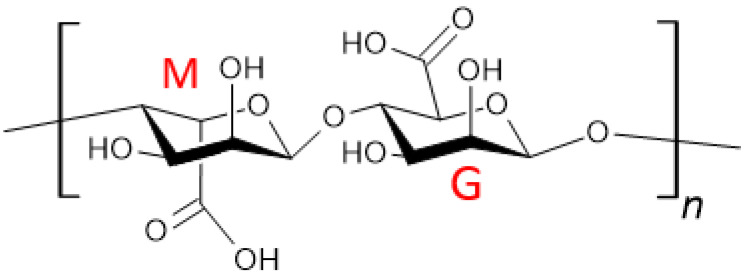
Chemical structure of alginate with highlighted monomers.

Alginate (see Figure 1) is a linear polymer of natural origin. It is composed of L-guluronic acid (G blocks) and D-mannuronic (M blocks) monomers linked by 1,4-glycosidic bonds (Figure 1). The ratio of the two mers varies depending on the polymer source [8].

The length of the M and G blocks affects the molar mass of the alginate and its physical properties [9]. A higher M/G ratio results in greater flexibility, while a lower M/G ratio increases brittleness [10]. Alginate can be of either plant or bacterial origin [11,12]. Plant-derived alginate is most commonly extracted from brown algae (*Phaeophyceae*), such as *Laminaria hyperborea*, *Laminaria digitata*, *Laminaria japonica*, *Ascophyllum nodosum*, and *Macrocystis pyrifera* [13]. In marine plants, it can constitute up to 40% of their dry weight, and likely serves a role analogous to cellulose [14]. Bacterial alginates are produced only by bacteria of the genera *Pseudomonas* and *Azotobacter* [10,15]. The molar mass of plant-based sodium alginate ranges from 32,000 to 400,000 g/mol [13]. The polymer has an affinity for multivalent metal cations, which results in its gelation. Protons also induce this process [16]. Gelation of alginate occurs through ionic crosslinking, covalent crosslinking, photo crosslinking, click chemistry reactions, thermal gelling, or cryogelation. Alginate can be chemically modified to enhance its properties. For example, the incorporation of peptides such as arginine-glycine-aspartic acid promotes cell adhesion and proliferation [13]. Carboxyl groups in alginate can be amidated, and hydroxyl groups can be sulfated. In addition, both groups can be esterified [17]. Sulfation of alginate may be aimed at producing a heparin mimetic. The derivatives obtained can be used as a drug or biomaterial—a drug carrier [18]. This biomaterial is considered non-toxic and biodegradable [10]. It has been reported that alginate exhibits antioxidant activity (reduces ferric ions) and anti-inflammatory properties, and can neutralize reactive oxygen species (ROS) [10,19]. In dentistry, alginate is primarily used for making dental impressions. Its main advantages are low cost and ease of preparation [20]. Alginates are widely used in wound dressings, drug delivery systems, and tissue engineering applications [17]. Some isolated case reports mention allergic contact dermatitis or respiratory irritation linked to alginate materials. However, true allergic reactions to pure alginate are very uncommon [21].

### 3.2. Cellulose

**Figure 2:**
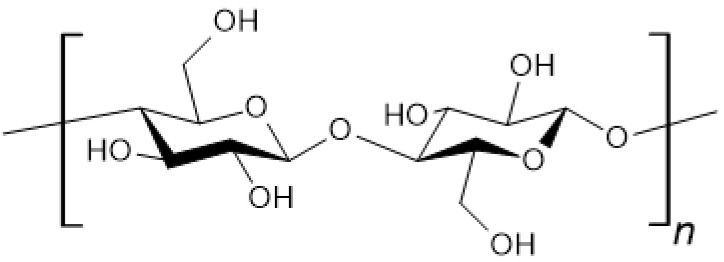
Chemical structure of cellulose.

Cellulose (Figure 2) is a linear polymer made of D-glucose mers linked by β-1,4-glycosidic bonds. It is the most common natural polymer on Earth [22,23].

Natural cellulose is extracted from plant sources such as wood and cotton. It can also be obtained from bacteria, fungi, and animals—tunicates [24,25]. Bacterial cellulose is easier to purify because it is not mixed with similar molecules, such as lignin or hemicellulose. Despite having the same chemical structure as celluloses of other origins, the bacterial polymer has slightly different physical properties. Among other things, it can absorb water to form hydrogels, which degrade more rapidly than plant-derived cellulose [25,26].

Cellulose can be chemically modified through its hydroxyl groups, resulting in various derivatives with altered physicochemical properties, such as solubility [25,27].

Cellulose can exist in the form of four allomorphs. Changing the crystalline structure is possible with suitable conditions of thermochemical preparation of the polymer [28]. Allomorphs differ in the mutual arrangement of cellulose chains and hydrogen interactions between molecules [26].

Cellulose has been proposed as a dressing material, but it does not exhibit antibacterial properties. Inhibition of microbial growth can be achieved by the addition of other compounds, such as octenidine or minocycline [29,30]. Another application is in the broad field of tissue engineering. This includes scaffolds for bone repair, where cellulose-based composites can release substances that induce osteogenesis [31]. Cellulose is also proposed as a material to replace ear cartilage; its mechanical properties make it a suitable candidate [32]. Using 3D bioprinting, it was possible to obtain a biomaterial based on cellulose and alginate, which was used to print a small model of an ear. Human chondrocytes bioprinted into the bioink had a high survival rate, providing hope for a new method of cartilage regeneration [33].

### 3.3. Chitosan

**Figure 3:**
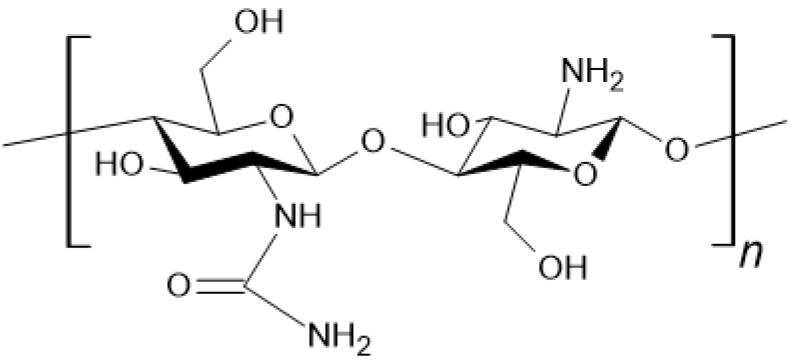
Chemical structure of chitosan.

Chitosan (Figure 3) is a linear polysaccharide, a deacetylated derivative of chitin. It is naturally found in the cell walls of fungi [34].

Chitin is the second most abundant polymer in nature after cellulose. Chitosan is the only naturally occurring cationic polysaccharide. The polymer consists of D-glucosamine and N-acetyl-D-glucosamine. Its molar mass ranges from 10 to 1000 kDa, influencing its properties [35]. Chitosan exhibits hemostatic, antibacterial, and antifungal effects. Its antimicrobial activity is based on amine groups. After protonation, it acquires a positive charge and interacts with the negatively charged bacterial surface, resulting in cell wall damage [36,37]. Another proposed mechanism involves chitosan penetrating bacterial cells, where it binds to DNA, inhibiting transcription and consequently disrupting translation [34,38]. However, studies have reported that medium molecular weight chitosan (9.0 × 10^4^ Da) can promote *E. coli* growth. Additionally, low concentrations of this biomaterial exhibited effects on the bacteria in vitro [39]. Chitosan’s antifungal activity depends on the fungal developmental stage. Additionally, its efficacy is influenced by the product batch, molar mass, and origin [40]. Chitosan is insoluble in water and most organic solvents. It dissolves in dilute acid solutions after the amino groups have been protonated [34,41]. Chitosan can be chemically modified. An example is 6-O-sulfated chitosan, which promotes neuronal differentiation of embryonic stem cells [42]. Chitosan is biodegradable. Its biocompatibility makes it suitable for tissue engineering [43], both for wound dressings to promote skin regeneration, aiding the healing of burns, ulcers, and post-operative wounds, as well as in dentistry to aid in the regeneration of periodontal (gum) tissues that have been damaged by disease or surgery. This biomaterial has low stiffness and strength, but moisture enhances these properties [38]. A notable property of chitosan is its blood–brain barrier permeability. Because of its mucoadhesion and permeability, it is being studied for drug delivery to the brain [44].

### 3.4. Collagen

**Figure 4:**
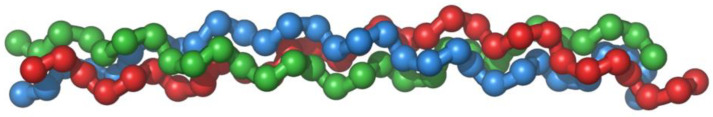
Collagen triple helix.

Collagen is one of the most common proteins in the extracellular matrix [45]. It occurs in the skin, bones, tendons, cartilage, and ligaments [46]. It accounts for approximately 30% of the total protein content in the human body. Twenty-nine collagen subtypes have been described, differing in composition and structure. Collagen is composed of a triple alpha helix. The chains of amino acids that build the helix can be different or identical [47]. It consists of 19 amino acids, with proline and glycine being the most abundant. Notably, it lacks cysteine; instead, it contains hydroxyproline, which is specific to this protein [48]. The best described is the most common type I collagen (Figure 4) [47].

Collagen is biocompatible, biodegradable, and has low immunogenicity [46]. However, in a small group of patients, it can induce an adverse immune response [49]. Such side effects can often result from inadequate purification of the material. Unremoved cellular debris, such as oligosaccharides or genetic material, is the most common cause of an immune response [50]. Collagen can be derived from animal sources or produced recombinantly [46]. It is degraded by collagenases. Methods to regulate the rate of degradation have been described in the literature [51].

Collagen scaffolds can be produced in two main ways. The first involves decellularization, which removes cells while preserving the collagen matrix and maintaining the original shape of the tissue. The second method extracts pure collagen to fabricate a functional scaffold [46]. Collagen is also used as a dietary supplement and as an ingredient in intra-articular injections for treating joint diseases. Although evidence regarding its effectiveness in cartilage disorders is limited, some studies suggest a potential beneficial effect, such as improving joint mobility, reducing pain, and supporting cartilage repair by stimulating the production of extracellular matrix components [52]. Clinical trials investigating joint microfracture procedures in combination with collagen matrix administration are also underway [53].

### 3.5. Cyclodextrins

**Figure 5:**
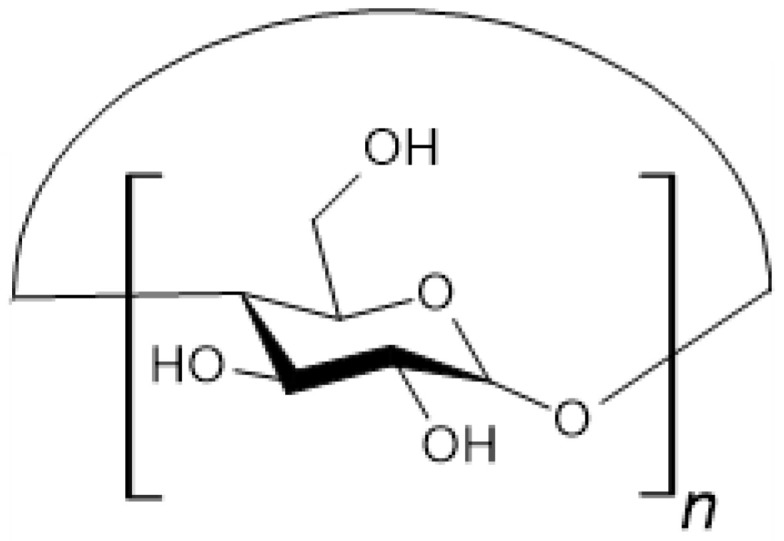
Chemical structures of: α-cyclodextrin (n = 6), β-cyclodextrin (n = 7) and γ-cyclodextrin (n = 8).

Cyclodextrins are a group of cyclic oligosaccharides composed of D-glucose subunits linked by α-1,4-glycosidic bonds. Three of the most well-known compounds are α-cyclodextrin (α-CD), β-cyclodextrin (β-CD), and γ-cyclodextrin (γ-CD) (Figure 5). They consist of six, seven, and eight glucose units, respectively. Cyclodextrins with a higher number of D-glucose subunits have also been described in the literature [54,55]. Larger compounds are characterized by greater flexibility [56].

Cyclodextrins are considered non-toxic when administered orally [57]. They are stable in water and certain organic solvents. However, at low pH, they undergo hydrolysis, which results in the formation of linear polymers and glucose. Interestingly, β-CDs are significantly less soluble than α-CDs and γ-CDs, most likely due to their molecular rigidity [58].

Cyclodextrins have a characteristic conical-shaped structure with a hydrophobic cavity and a hydrophilic outer surface [59,60]. This structure arises from the chair conformation adopted by the glucose rings. Active substances can be placed inside the cavity of cyclodextrins. Van der Waals forces and hydrophobic interactions are mainly responsible for the formation of inclusion complexes. Electrostatic and hydrogen bonds are not the dominant interactions, they also affect the binding of the active substance. Several drugs utilizing cyclodextrins as carriers for active substances have already been approved for clinical use [61].

Cyclodextrin derivatives can be synthesized by introducing functional groups into their rings. Chemical modification of secondary and primary hydroxyl groups aims to enhance their solubility, bioavailability, and stability. Additionally, other polymers or active substances can be conjugated to cyclodextrins, allowing for the modulation of complex stability and drug release rates [62].

### 3.6. Gelatin

Gelatin is a biomaterial derived from the partial acid or alkaline hydrolysis of collagen [63,64]. Due to denaturation, a linear polymer is formed [64]. It consists of a mixture of molecules of different weights. The α, β, and ɣ chains have molecular masses of 80–125 kDa, 160–250 kDa, and 240–375 kDa, respectively [65]. Amino acids included in its composition are proline, glycine, and hydroxyproline. By origin, we distinguish between gelatin from mammalian (usually bovine), marine, and poultry sources [66]. Due to its origin, gelatin has properties similar to collagen [67]. It is biocompatible, non-immunogenic, biodegradable, and non-toxic [68,69]. The disadvantages of this biomaterial are poor mechanical properties and low thermal stability. To address these limitations, various crosslinking methods are employed. However, the use of chemical reagents for this process can increase the toxicity of gelatin [70]. Physical crosslinking methods, such as UV radiation or microwave treatment, are also used; however, they are less effective and more difficult to control than chemical methods. The described biomaterial is used as a drug delivery system and in tissue regeneration [64]. Gelatin naturally contains tripeptide sequence (Arg-Gly-Asp) motifs, which increase cell adhesion [71].

In dentistry and surgery, hemostatic preparations based on gelatin are used. Gelatin systems are commercially available. They are used in inhibiting postsurgical bleeding. They are bioabsorbable and well tolerated by the human body [72]. In addition, gelatin is proposed as a dressing material and drug delivery system [67]. Since gelatin lacks intrinsic antibacterial properties, bacteriostatic additives are incorporated to prevent microbial growth. Gelatin-based dressings have been shown to improve wound healing. Three-dimensional printing technology has been explored to customize dressings for better wound conformity [73].

### 3.7. Hyaluronic Acid

**Figure 6:**
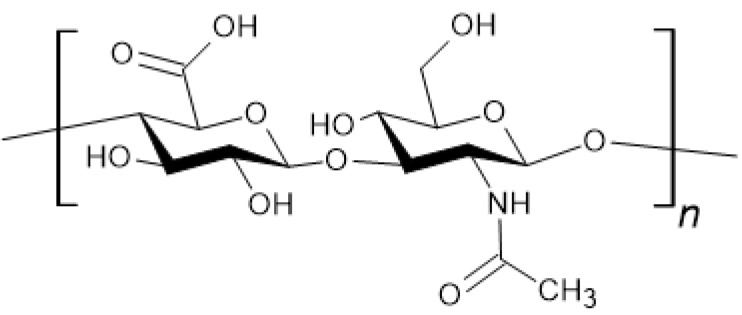
Chemical structures of hyaluronic acid.

Hyaluronic acid (Figure 6) is a polymer (glycosaminoglycan) composed of D-glucuronic acid and N-acetyl-D-glucosamine subunits linked by β-1,4- and β-1,3-glycosidic bonds. The molar mass of this compound varies significantly and depends on its function in the human body. High-molecular-weight hyaluronic acid can be cleaved into smaller molecules by the enzyme hyaluronidase in vivo [74,75]. This compound is a major component of the extracellular matrix [76]. It could bind to proteins, lipids, polysaccharides, and other molecules. It is biodegradable and biocompatible [77]. Hyaluronic acid is hydrophilic; it can absorb large amounts of water, increasing its volume up to 1000 times and forming a hydrated matrix [78]. This polymer is widely distributed in nature. It occurs in both bacteria and eukaryotes. It is produced in large quantities during tissue damage and repair. It modulates angiogenesis and processes occurring during inflammation [79,80]. The biological effects of hyaluronic acid depend on its molecular weight: low-molecular-weight hyaluronic acid stimulates endothelial cell migration and proliferation, whereas high-molecular-weight polymers exhibit the opposite effects [74]. Industrially, it is obtained by two routes: extraction from animal tissues and microbial production [81]. Hyaluronic acid can be chemically modified: conjugated and cross-linked. Cross-linking is primarily used to form hydrogels and alter physicochemical properties, while conjugation enables the attachment of active substances that are released during polymer degradation [78]. The most well-known application of hyaluronic acid is in dermatology and cosmetology, where it is used as a wrinkle filler and to promote tissue regeneration. In addition to its filling properties, it stimulates the in vivo synthesis of type I collagen and elastin [78,82]. In dental surgery, it has been used successfully, among other things, to inject the temporomandibular joint to treat its disorders. Numerous studies demonstrate a reduction in pain symptoms in patients treated with injections [83].

Hyaluronic acid could also be present in products designed for post-procedural oral hygiene. It supports angiogenesis, inhibits the activity of pro-inflammatory cytokines, and reduces cellular infiltration, thereby minimizing swelling and pain [84]. Additionally, it may help limit microbial growth by physically shielding the wound, forming a thin, flexible protective layer that safeguards the tissue from external factors. Hyaluronic acid also alleviates pain, dryness of the mucous membranes, and burning sensations [85].

### 3.8. Hydroxyapatite

Hydroxyapatite is an inorganic biomaterial with the chemical formula: Ca_10_(PO_4_)_6_(OH)_2_ [86]. It occurs naturally in the bones and teeth of vertebrates. In humans, the size of hydroxyapatite crystals in bones changes with age. Under pathological conditions, it can precipitate in other organs [87]. Hydroxyapatite as a biomaterial can be of natural or synthetic origin. It has a wide range of applications in tissue engineering [88,89]. Naturally derived hydroxyapatite can be obtained from animal-derived waste, such as eggshells and bones [88]. In addition, in humans, hydroxyapatite is a key component of bone, enamel, and dentin [90,91]. This biomaterial can be extracted from plants such as red marine algae [92]. The advantage of natural hydroxyapatite is the content of additional trace elements such as zinc, magnesium, fluorine, silicon, and strontium in its structure. These elements enhance the biological properties of the material [89]. For example, the incorporation of divalent iron cations results in increased osteoblast activity, without increasing cytotoxicity [93]. The addition of strontium reduced the cytotoxicity of hydroxyapatite, while the simultaneous addition of strontium and fluorine increased cell proliferation [94,95]. Hydroxyapatite is used as a bone substitute [96]. The advantages of this biomaterial are osteoconductivity and biocompatibility. Low fracture toughness and a lack of antimicrobial properties make it necessary to use composites and functionalize the biomaterial in certain cases. Hydroxyapatite nanoparticles, defined as particles with at least one dimension below 100 nm, are also utilized in medicine. Their small size results in a significantly increased surface area, which enhances bioactivity. Nanoparticles promote bone growth and are non-toxic at appropriate doses; however, at elevated concentrations, they may exhibit cytotoxic effects [97]. Hydroxyapatite and its nanoparticles are also proposed as drug carriers. The Zeta potential of nanoparticles can be either positive or negative, and modifying this parameter allows for the optimization of pharmacokinetics [98].

Nanohydroxyapatite has been gaining increasing importance in recent years in the field of dentistry. It is known for its excellent anti-caries properties. By mimicking the natural mineral structure of tooth enamel it helps to repair and strengthen damaged areas. Nanohydroxyapatite can also reduce tooth sensitivity and protect against further decay. Its biocompatibility and effectiveness make it a promising alternative to traditional fluoride treatments [99].

### 3.9. Silk

Silk is a fiber composed primarily of proteins, with small amounts of lipids and polysaccharides [100]. Its main constituent proteins are fibroin and sericin. Sericin functions as a coating that binds fibroin fibers together [101]. The most common silk-producing organisms are silkworms and spiders [102]. Due to the difficulties of mass spider breeding, silkworms are now the main source of silk. Recently, silk produced by bioengineered bacteria has gained popularity due to the simplicity of bacterial culture, its reproducibility, and the short production time required [103]. Among bacterial hosts, *E. coli* is the most commonly used because of its ease of cultivation [104]. Silk is currently being tested as a drug carrier for cancer therapy. The substances carried can be of synthetic (e.g., cisplatin) or natural (e.g., curcumin) origin. Noteworthy is the labeling of silk nanoparticles with peptides targeting cancer cells [105]. Genetic engineering techniques have been proposed to introduce additional functional proteins into silk’s structure, potentially enhancing its biomedical applications [103]. Silk is biocompatible and non-toxic. Under physiological conditions, it degrades slowly. Scaffolds based on this material are compared to collagen due to its good tolerance by the body [106]. Silk does not exhibit antibacterial activity. This is a disadvantage, but it can be reduced by including particles with antimicrobial activity in scaffolds [107]. Silk can also be combined and cross-linked with other proteins, polysaccharides, and polymers. For example, the addition of collagen increases the stiffness of the resulting composite [108]. In dentistry and dental surgery, silk is used for sutures. However, silk sutures have a disadvantage—biofilm can accumulate on them, causing secondary infections [109].

### 3.10. Comparison of Biomaterials

Table 1 provides a comparative analysis of the natural biomaterials described above, commonly used in dental and medical applications. It highlights key aspects, such as clinical effectiveness, limitations, cost-effectiveness, and the level of available evidence for each material, along with their main applications. This overview helps in understanding the suitability of these biomaterials for different therapeutic and regenerative purposes.

## 4. Natural Biomaterials in Selected Areas of Stomatology

Dentistry has been advancing at an increasingly rapid pace. The development of new biomaterial formulations is necessary, as no universal material suitable for all applications has been found. Demand in this market continues to grow, along with increasing life expectancy and public awareness of dental health.

Natural biomaterials in dentistry are used for both disease prevention and treatment. Their diverse physical, chemical, and biological properties allow each biomaterial to serve different applications. In this regard, this paper seeks to analyze the existing literature, describe selected natural biomaterials, and highlight their applications in periodontal disease, caries, and implantology.

### 4.1. Periodontal Disease Prevention and Regeneration

Periodontal diseases are a set of inflammatory disorders involving the tissues surrounding the teeth, including the gums, periodontal ligament, and alveolar bone. Initial gingivitis, caused by bacteria in plaque, can progress into chronic periodontitis. Without treatment, it leads to loss of gum tissue and gum recession [110]. Bacterial plaque control slows or stops the development of the disease. Control of the growth of plaque-forming bacteria can be carried out using substances with antimicrobial activity. Conventional therapies, such as the use of mouthwash or toothpaste, are characterized by a short duration of action and low penetration of the periodontal pocket. Therefore, polymeric drug delivery systems offer an advantage by extending the release of active substances and ensuring localized action in the oral cavity, minimizing systemic side effects in the gastrointestinal tract [111]. In addition to bacterial involvement, the background of the disease is more advanced. It involves genetic and environmental factors, including oxidative stress and chronic inflammation [112].

**Figure 7:**
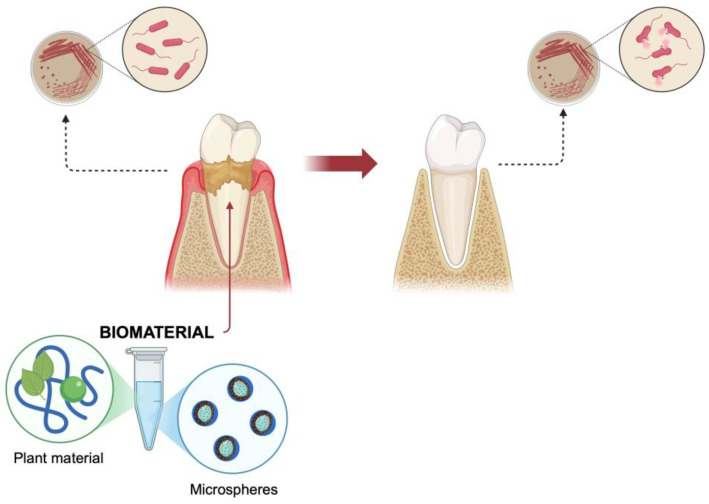
Periodontal disease prevention and regeneration scheme.

According to statistics, gum disease affects more than half of the population [113]. Some data even suggest as high as 90% of the population [114]. Due to the frequency and global nature of the disease, the development of new formulations would reduce the health and economic consequences. Prevention and treatment of periodontal diseases may include the use of drug delivery systems that release substances with therapeutic properties (Figure 7).

Scholz et al. (2017) described a low-cost technique for producing alginate microspheres that release chlorhexidine. The crosslinking agent was calcium chloride, and the beads were obtained by the dripping method. The chlorhexidine release profile could be adjusted by changing the size of the beads and the concentration of the active ingredient, among other factors [115].

A novel formulation based on resveratrol and cyclodextrins was developed by Paczkowska-Walendowska et al. (2021). Several polyphenol systems with different cyclodextrins were created. The best system was based on resveratrol and β-cyclodextrin. It was characterized by strong mucoadhesive properties that ensured long-lasting release of antioxidant and anti-inflammatory polyphenols at the route of administration [112].

In addition to individual active substances, it is possible to use broad-spectrum plant extracts.

Chitosan is considered a carrier with excellent mucoadhesion. For this reason, it is a promising candidate for developing a drug delivery system [104].

A mucoadhesive drug delivery system containing a chitosan-based extract of *S. baicalensis radix* was characterized by prolonged release of polyphenols. In addition, it inhibited hyaluronidase activity, which may promote periodontal tissue regeneration [116]. Another approach involves 3D printing of the final product. The 3D-printed chitosan-based hydrogel scaffolds carried the previously mentioned extract. The release of baicalein was investigated, which was prolonged compared to the extract alone. The formulation also inhibited hyaluronidase and was biocompatible. In addition, it had a positive effect on fibroblast proliferation in an in vitro wound healing assay [117].

Microspheres based on alginate and chitosan were developed by Park et al. (2005). They were loaded with minocycline. These microspheres released the antibiotic directly at the treatment site for up to seven days and demonstrated efficacy against *Prevotella intermedia* [118].

Few studies describe the use of silk in the treatment of periodontal disease. Resveratrol-loaded silk fibroin nanoparticles have been well characterized. They were obtained by incubating resveratrol with silk fibroin nanoparticles. They were first tested on the RAW 264.7 mouse macrophage cell line. In basal conditions, they promoted macrophage activity; however, when cells were stimulated with lipopolysaccharide (LPS), the nanoparticles inhibited the inflammatory response [119]. In a rat model of diabetes with periodontitis, the nanoparticles reduced gingival inflammation, highlighting their potential therapeutic benefits [120].

Imber et al. (2021) studied the effect of collagen scaffolding on periodontal regeneration. The study conducted on dogs showed that the scaffold was biocompatible and had a beneficial effect on bone growth and periodontal soft tissues [121]. Similarly, membranes were also described by Ashworth et al. (2018). The membranes were made by lyophilizing a collagen suspension in hydrochloric or acetic acid. They were then cross-linked with 1-ethyl-3-(3-dimethylaminopropyl) carbodiimide hydrochloride and N-hydroxysuccinimide. The study investigated how the size of the pores and their arrangement in the scaffold affect the migration of human fibroblast cells of the periodontal ligament. The proper orientation of the pores and their location were shown to affect cell invasion in the scaffold. The best results were achieved for pores with a diameter of 100 µm. Migration efficiency was weakest for pores arranged randomly. The results indicate that it is possible to influence the rate of colonization of the scaffold by cells by changing biomaterial porosity [122].

A complex system based on gelatin nanofibers was described by Wang et al. (2019). It included inhibitors of protein kinases involved in the expression and transcription of metalloproteinases. The inhibitors were loaded into poly(ethylene glycol)-block-polycaprolactone micelles, which were then electrospun with an aqueous gelatin solution. To prolong the drug release period and reduce the solubility of the gelatin, the nanofibers were cross-linked with 1-(3-dimethylaminopropyl)-3-ethylcarbodiimide hydrochloride and N-hydroxysulfosuccinimide. The CCK-8 test showed no cytotoxicity of the formulation. In a study on dogs with LPS-induced periodontal disease, it was found that the formulation inhibited connective tissue matrix degradation and promoted bone regeneration [123].

Nguyen et al. (2021) conducted a human study on periodontal disease treatment using subgingival application of 0.2% hyaluronic acid gel. Periodontal pockets were irrigated with NaCl solution, dried, and filled with 1 mL of gel. Periodontal evaluation after six weeks showed a reduction in pocket depth [124]. Mohammad et al. (2023) also described the effect of subgingival administration of 0.8% high molecular weight hyaluronic acid gel. The gel was introduced into periodontal pockets until they were completely filled. The pockets were covered with a dressing for one week. Improvements in gingival and periodontal conditions and a reduction in inflammatory biomarkers were observed. Compared to scaling and superficial root cleaning, the administration of the gel appeared to be more effective in reducing pro-inflammatory cytokines [125].

Tabary et al. (2014) described a drug delivery system based on paper points. Cellulose membranes were oxidized to increase the number of carboxyl groups. The membranes were then functionalized with β-CD or maltodextrin using the pad-dry-cure textile finishing method. The resulting drug delivery system was loaded with chlorhexidine. The obtained membrane was found to be non-toxic, but in vitro cytotoxicity increased after loading with antiseptic. Tests in human plasma confirmed the prolongation of chlorhexidine release [126].

Cellulose derivatives can be used as physical barriers to create a suitable environment for periodontal tissue regeneration. Fornazier et al. (2021) developed a cellulose acetate-based membrane using the casting method. Additives such as calcium glycerophosphate and sodium carboxymethyl lignin improved water absorption, and the membranes exhibited asymmetric pore distribution, suggesting their suitability for dental applications [127].

Johnson et al. (2020) developed a hydrogel system containing cellulose nanofibers (CNFs) and κ-carrageenan oligosaccharide (CO) nanoparticles. The system carried the antimicrobial substances—surfactin and Herbmedotcin. The N,N′-methylenebisacrylamide cross-linked hydrogel contained CO-CNF nanoparticles prepared according to the method described in an earlier publication. The resulting material was shown to exhibit strong antibacterial activity against *Streptococcus mutans*, *Porphyromonas gingivalis*, *Fusobacterium nucleatum*, and *Pseudomonas aeruginosa*. The obtained material was characterized by antioxidant properties, which most likely resulted from the synergistic action of its individual components. Testing on human gingival fibroblasts showed that the hydrogels had anti-inflammatory and antioxidant effects. In a biofilm formation test, they inhibited bacterial growth [128].

Berta et al. (2021) formulated a resveratrol-based mouth spray using 2-hydroxypropyl-β-cyclodextrin. The formulation was tested on children aged from 2 to 5 years with plaque-induced gingivitis. The spray was applied once a day after tooth brushing for 14 days. Results indicated reduced plaque accumulation and gingival inflammation. The described spray is an alternative to oral hygiene liquids containing chlorhexidine, as this substance is not recommended for children under the age of 6 due to the risk of swallowing the liquid [129].

Cyclodextrin complexes that carry resveratrol and its analogs were characterized by Lim et al. (2020). Inclusion complexes were prepared by dissolving resveratrol, pterostilbene, oxyresveratrol, or piceatannol in a solution of 2-hydroxypropyl-β-cyclodextrin, which acted as a solubilizer. Resveratrol analogs have been tested due to their longer half-life. In a test of antimicrobial properties, complexes with pterostilbene showed the greatest activity against *Fusobacterium nucleatum*. The described complexes also caused leakage of bacterial cellular contents. Complexes with pterostilbene also exhibited anti-inflammatory properties in a test on RAW 264.7 cells. Additionally, these complexes showed anti-inflammatory properties by reducing pro-inflammatory cytokine levels and inhibiting the NF-κB pathway [130].

### 4.2. Dental Caries Prevention

Tooth decay is the localized destruction of a tooth’s hard tissues caused by bacteria. The demineralization of the tooth is caused by metabolites produced by microorganisms during carbohydrate fermentation [131]. Caries is a multifactorial disease and can affect both deciduous and permanent teeth [132]. In addition to aesthetic defects, the disease also impacts patients’ lives. In its advanced stage, it can lead to tooth loss. It affects most adults, and its incidence is steadily increasing [133].

The caries process occurs in multiple stages, beginning with the accumulation of dental plaque on the tooth surface. When bacteria in the plaque metabolize sugars, they produce acids like lactic acid, leading to a drop in pH below the critical threshold of 5.5. This acidic environment causes the loss of calcium and phosphate ions from enamel, resulting in demineralization. Early-stage carious lesions, visible as white spot lesions, represent subsurface mineral loss and are still reversible through remineralization supported by saliva and fluoride. However, if demineralization persists and outweighs remineralization, enamel breakdown progresses to cavitation, requiring professional dental intervention [134].

Caries is a chronic disease that usually progresses slowly. Its progression depends on the balance between remineralization and demineralization [131]. Current fluoride-based prophylaxis can inhibit enamel demineralization. However, it does not prevent biofilm formation and can also cause side effects related to excessive fluorine intake [135].

**Figure 8:**
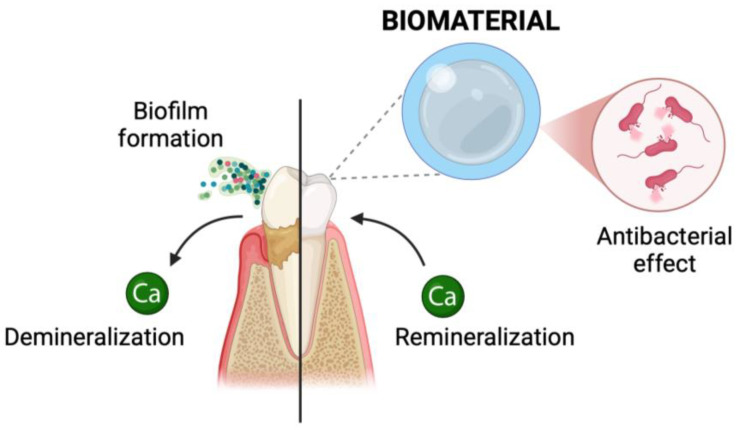
Dental caries prevention scheme.

Current preventive strategies include maintaining proper oral hygiene (such as brushing, flossing, and rinsing), using fluoride, sealing pits and fissures (the most susceptible areas, the shape of which promotes plaque formation and buildup), and using xylitol (reduces adhesion and the number of selected bacteria). Additionally, the use of erythritol, glycine, and bicarbonate powders has become an effective approach in combating dental biofilm. Erythritol inhibits bacterial adhesion and disrupts biofilm formation, glycine provides gentle abrasion to remove plaque without harming enamel, and bicarbonate helps neutralize acids and scrub tooth surfaces, contributing to a cleaner, healthier mouth [136]. Due to the transmissible nature of tooth decay, there is a correlation between the amount of caries-causing bacteria in the mouth of children and their parents. Therefore, treatment of this disease in parents is important in terms of caries prevention in their children (Figure 8) [137].

Jiang et al. (2024) described nanoparticles based on antimicrobial chitosan and sodium phytate that slow down enamel demineralization. The nanoparticles were created using an ionic gelation method. Activity against *S. mutans* was investigated. The formulation exhibited greater antibacterial properties than chitosan alone. In an in vitro test, the nanoparticles inhibited enamel demineralization. In animal studies, the formulation was shown to slow caries development without disrupting the microbiota or exhibiting inflammatory properties [135].

Samprasit et al. (2015) developed mucoadhesive electrospun nanofiber mats based on the thiol derivative of chitosan. The formulation carried *Garcinia mangostana* extract. The biomaterial exhibited antimicrobial properties against *Streptococcus mutans* and *Streptococcus sanguinis*. Higher extract content increased the antibacterial effect. The mats were characterized by their biocompatibility. In an in vitro study on human keratinocytes and gingival fibroblast cells, they showed no cytotoxicity. According to the researchers, the mats can be safely used for at least 60 min. In an in vivo volunteer study, the biomaterial reduced bacterial levels in the subjects’ saliva and exhibited long-term adhesion in the oral cavity [138].

A nanocomposite material based on nanocellulose, green alga *Ulva lactuca* extract, and silver nanoparticles was developed by Hamoud et al. (2023). The nanocellulose was produced from algae harvested from the Red Sea. The nanoparticles were obtained by mixing a solution of silver nitrate, nanocellulose, and alga extract. The resulting formulation was then enriched with fluoride. The resulting composite had a crystalline structure and a fibrous surface. A comparison between fluoride-containing and fluoride-free nanoparticles showed that the enriched formulation exhibited stronger antibacterial activity against *S. mutans* and *L. acidophilus*. The authors indicate that the resulting composite can be tested as a component of toothpaste or as an additive for dental fillings [139].

Silvestre et al. (2023) developed drug delivery systems based on chitosan and alginate for curcumin delivery in photodynamic therapy applications. Nanoparticles were produced by polyelectrolyte complexation, and buccal films containing the nanoparticles were prepared by solvent casting. Release studies showed that the film formulation released curcumin faster than nanoparticles alone, while both systems prolonged drug release overall. In vitro testing revealed no antifungal activity against *Candida albicans*. However, after light activation, both curcumin-loaded and unloaded nanoparticles demonstrated antibacterial effects against *Streptococcus mutans*. Curcumin solution itself showed antibacterial properties even without light activation. Although the new formulations successfully controlled curcumin release and showed potential for antibacterial applications, further work is needed to optimize their effectiveness in photodynamic therapy [140].

To combat caries, Bright et al. (2022) described a dual-action hydrogel system. It worked by releasing nitric oxide and fluoride. The nitric oxide (NO) donor was S-Nitrosoglutathione (GSNO). Hydrogels were prepared by dissolving sodium alginate, Pluronic F127, GSNO, and sodium fluoride. Solutions with varying substrate concentrations were poured into Petri dishes and incubated at 37 °C. The hydrogels were then cross-linked with calcium chloride. The procedure was completed by cutting the gel and rinsing and drying it. The obtained hydrogels had a porous structure. Under physiological conditions, the biomaterial released NO and fluoride in a controlled manner. Antimicrobial properties were confirmed against *E. Coli* and *S. mutans*. In an enamel model, the hydrogel was shown to prevent hydroxyapatite demineralization. In vitro studies on HGF and hFOB 1.19 cell lines showed a lack of cytotoxicity. The resulting hydrogels were biocompatible, inhibited enamel demineralization, and exhibited antibacterial properties [141].

Cellulose derivatives have also been used to inhibit the formation of enamel white spot lesions. To treat the onset of caries, Zhang et al. (2021) created a mineralization film based on hydroxycellulose (HPMC). The initial stage of the study involved the synthesis of AFPC nanoparticles. They were prepared by mixing a solution of calcium chloride and poly-L-aspartic acid with a solution of sodium bisulfate and sodium fluoride. The pH of the resulting solution was raised and the precipitate was separated and ground to obtain AFPC. The mineralization film was prepared by evaporating the solvent from the HPMC solution along with the AFPC nanoparticles. A study on extracted human teeth demonstrated that the formulation could reduce tooth discoloration and mineral loss due to pH fluctuations. In addition, the film can promote enamel mineralization. The authors concluded that their product could be applied by patients overnight, allowing it to adhere to the enamel surface for several hours [142].

An attempt to develop a formulation to prevent caries was also made by Garcia et al. (2023). They developed chitosan microparticles that encapsulated essential oils and were produced via spray drying. Chitosan was dissolved in acetic acid, and then geranium and lemongrass oils were added. The mixture was homogenized and sonicated and then subjected to spray drying. The formulation showed a stronger effect against *C. albicans* than *S. mutans*. Essential oils showed cytotoxicity against RAW 264.7 cells, but their encapsulation in chitosan reduced this effect. Chitosan microparticles with essential oils may provide an alternative treatment for caries caused by mixed biofilms of *C. albicans* and *S. mutans* [143].

### 4.3. Bone Substitutes in Implantology

Tooth loss is associated with irreversible bone loss, which causes problems for subsequent implant treatment. Successful implant placement requires adequate bone dimensions. One solution may be a bone graft or the use of a bone substitute to promote its reconstruction [144]. The bone fragment can be transplanted from a donor to a recipient; both allogeneic and autogenous transplantations are possible. Materials of plant or animal origin are also used. In addition, synthetic materials are available [145]. Ideal bone substitutes should be characterized by osteoinduction, osteoconduction, osteogenesis, and osteointegration [146].

An example of plant-derived material is AlgiPore^TM^. It is hydroxyapatite derived from marine algae—*Corallina officinalis*. AlgiPore^TM^ is obtained by pyrolysis and autoclaving. In preclinical in vitro and in vivo studies, this biomaterial has been shown to promote bone development. This material is used by mixing hydroxyapatite granules with blood to form a paste-like consistency, which is then used to fill the bone defect [147].

Animal-derived hydroxyapatite has been proposed as a possible bone graft. Material produced from bovine teeth was described by Ratnayake et al. (2020). The hydroxyapatite was obtained by cutting the teeth, deproteinizing, and defatting them. The material was chemically stable and showed minimal degradation after 28 days of incubation in simulated body fluid. Biological tests showed that hydroxyapatite is non-toxic, promotes Saos-2 cell line proliferation, and induces osteonectin expression, suggesting its potential osteoinductive properties. The developed material showed promise as a cost-effective and biocompatible biomaterial for bone regeneration [148].

An antibiotic-releasing material based on chitosan and hydroxyapatite was developed by Giordano-Kelhoffer et al. (2023). Doxycycline was chosen because of its broad spectrum of action. The new biomaterial was obtained by extruding chitosan fibers with the addition of hydroxyapatite and doxycycline. Chitosan was dissolved in acetic acid, followed by the addition of hydroxyapatite and the antibiotic. The mixture was then extruded through a needle into a NaOH solution, which served as the crosslinking agent. The study describes how the conditions for obtaining the biomaterial affected its physical properties. A limitation of the study was the lack of tests for cytotoxicity and how the material affects bone formation processes [149].

A hybrid material based on low-molecular-weight hyaluronic acid and carboxymethylcellulose was described by Lin et al. (2022). In the first step, the hyaluronic acid was fragmented using γ-rays. The resulting polymer had a mass of 200 kDa. Its effect was tested on dental pulp stem cell lines from healthy donors. It was shown that the small-molecule hyaluronic acid stimulated cell proliferation via mitogen-activated kinase (MAPK). An in vivo study on a rabbit model of bone loss examined how a hybrid biomaterial affected bone reconstruction. It was shown that the low-weight-molecule hyaluronic acid in the hydrogel exerted a stronger osteoregenerative effect than the high-mass hyaluronic acid [150].

Koike et al. (2019) described the use of bacterial cellulose as a carrier of bone morphogenetic protein 2 (BMP-2). Membranes of compressed cellulose were coated with BMP-2 solution, and the resulting material was tested in vivo on a rabbit frontal sinus model. Bacterial cellulose demonstrated excellent biocompatibility and could serve as both a barrier and a carrier for controlled drug release, making it an ideal biomedical material for dental surgery. Meanwhile, the addition of BMP-2 to the membrane stimulated cell proliferation, which translated into faster bone regeneration in rabbits [151].

A bone graft based on hydroxyapatite and collagen derived from sea cucumbers has been developed by Wahyuningtyas et al. (2019). Collagen extracted from *Stichopus hermanni* and hydroxyapatite were combined in a ratio of 35:65. The MTT assay on fibroblasts of the Vero cell line showed that the resulting biomaterial was not cytotoxic. The in vivo test on rats consisted of drilling a defect in the femur and filling it with the biomaterial. Normal collagen and collagen derived from *S. hermanni* were used as a comparison. It was shown that the combination of hydroxyapatite and collagen from sea cucumber had the best effect on bone regeneration. The highest number of osteoblasts and signs of active bone remodeling were observed in this group. The main limitation of the study was the very poor description of the biomaterial preparation method [152].

Lin et al. (2019) described the effect of a collagen-containing biomaterial on preventing bone resorption after tooth extraction in humans. In 51 patients after tooth extraction, the alveolus was filled with the biomaterial and sutured. The material used consisted of 30% porcine type I collagen. The other ingredients were hydroxyapatite and β-tricalcium. It was tested whether dental bone atrophy would occur after tooth extraction and filling the alveolus with biomaterial. Radiographic measurements of the alveolar height were taken before and after surgery. At 3 months after extraction, there were no statistically significant differences. Despite the small study sample, it was proven that the material used can be useful in preventing bone atrophy after tooth extraction [153].

Li et al. (2020) developed an injectable, thermosensitive hydrogel containing chitosan, gelatin, β-glycerophosphate, and erythropoietin to support maxillary sinus floor augmentation. The hydrogel transformed into a gel at body temperature and released erythropoietin in a controlled manner, promoting stem cell proliferation and osteogenic differentiation. In a rabbit model, it significantly enhanced bone formation compared to a drug-free material. The hydrogel was biocompatible and offered a minimally invasive alternative for sinus bone regeneration [154].

A retrospective evaluation by Cosola et al. (2022) describes the course of a sinus lift using collagen and subsequent implant placement. In 10 patients, the sinus bone was lifted using a matrix made of horse collagen. An apical bone fracture was also performed. The implant was placed after six months of healing. None of the patients registered serious problems. Histomorphological evaluation of one patient revealed complete biodegradation of the implanted biomaterial. The bone at the matrix implantation site had increased in size by 6 mm. The study indicates positive clinical outcomes of collagen application in bone reconstruction. However, the main limitation is the small number of cases studied [155].

### 4.4. Dental Implants Coating

**Figure 9:**
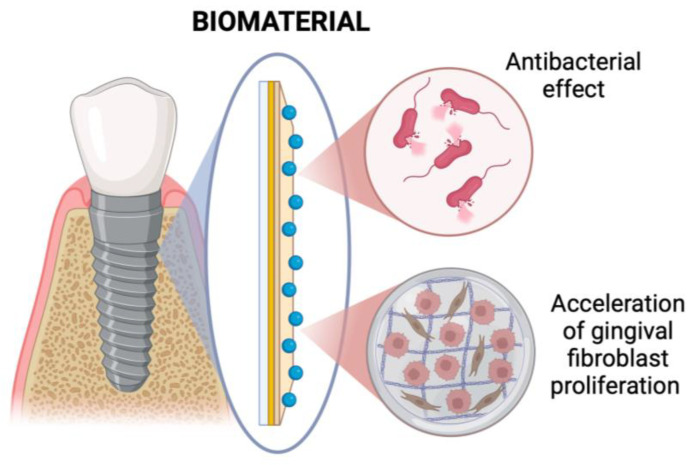
Dental implants coating scheme.

Biocompatible devices such as dental implants make it possible to replace missing teeth [156]. Cementless implants are an excellent alternative to dentures supported by teeth and soft tissues. Implant treatment is considered safe and effective. The material suitable for creating implants is titanium because of its biocompatibility and osteointegration. Threaded titanium implants have been successfully used clinically [156,157]. The topography of the implant affects the connection between the bone and the bolt. The screw surface should have the right roughness, both excessively large and too small roughness are unfavorable. The implant and tissue damage induce inflammation. During wound healing, the pro-angiogenic properties of the biomaterial are desirable. Due to the risk of bacterial growth on the surface of the implant, antibacterial properties will accelerate recovery (Figure 9) [158].

An antimicrobial implant coating based on a composite material was developed by Knopf-Marques et al. (2019). The coating consisted of gelatin and an aldehyde derivative of hyaluronic acid. The coatings were applied to the substrate by spin-coating. It was then enriched with poly(arginine). In the final step, the coating was cross-linked with the enzyme transglutaminase. Poly(arginine) is a polycationic polymer with antimicrobial and anti-inflammatory properties. The modified hyaluronic acid was to ensure its gradual release. The resulting coating was stable and provided prolonged release of the active ingredient. Tests confirmed the coating’s antibacterial activity against *Staphylococcus aureus*. It was also verified how the obtained formulation would affect human umbilical vein endothelial cells. The coating promoted the formation of intercellular connections and increased the expression of the angiogenesis marker VEGFA [158].

Atluri et al. (2024) developed a chitosan-based coating for titanium implants containing metronidazole. Compared to PLGA, chitosan coatings provided more uniform drug release, better antibacterial effects (against *Prevotella intermedia* and *Treponema denticola*), and enhanced proliferation and adhesion of induced pluripotent stem cells. They were also less cytotoxic. Low-molecular-weight chitosan further promoted BMP-2 expression, supporting osteogenesis [159].

Kim et al. (2022) developed a hydrogel implant coating based on gelatin (GelMA) and an antibacterial ginger extract. The hydrogel was applied to titanium surfaces, photocrosslinked, and lyophilized. Both the extract and its fraction showed antibacterial activity against oral pathogens (*S. mutans*, *P. gingivalis*), with the fraction proving more effective and selected for further studies. In vitro tests showed that with an appropriate fraction dose, the hydrogel remained biocompatible. In vivo studies in rats demonstrated that, after 6 weeks, implants with the hydrogel coating promoted better bone growth compared to controls. The key factor was the proper dosage of the ginger fraction [160].

Lin et al. (2021) developed a chitosan–zinc oxide (ZnO) coating for titanium implants. The hybrid coating, applied to etched titanium surfaces, showed enhanced antibacterial activity against *E. coli* compared to chitosan alone, likely due to zinc ion release. Biocompatibility tests on MG-63 cells confirmed that the coating was non-cytotoxic [161].

Wang et al. (2022) developed an implant coating based on chitosan, hyaluronic acid, and microRNA-21 to promote gingival fibroblast proliferation around implants. Nanoparticles carrying miR-21 were produced by ionotropic gelation and applied to titanium surfaces with gelatin, then freeze-dried. In vitro studies confirmed no cytotoxicity, efficient miR-21 transfection, and accelerated fibroblast proliferation. However, the coating showed no antibacterial activity [162].

Perpendicularly located collagen fibers called Sharpey’s fibers run between the alveolar bone and the tooth. Erturk et al. (2022) described a biomaterial that mimics these filaments. The fabrication of this material required the creation of a nanoporous mold, which was produced by anodizing. The homogenous pores in the mold were about 104 nm. The nanoporous collagen-gelatin film was prepared by dissolving the gelatin and collagen in water, adding poly(ethylene glycol), diglycidyl ether (crosslinking agent), adjusting the pH, and casting the gel into nanoporous molds. A study on osteosarcoma cell line (Saos-2) showed that films with increased roughness promoted cell adhesion and osteogenic differentiation [163].

A gelatin nanocomposite releasing curcumin was described by Dizaj et al. (2023). It would find application in coating healing abutment implants. The titanium surface was coated with a solution containing gelatin and nanocurcumin by dip coating. The crosslinking agent was glutardialdehyde. In the MTT test, the formulation was not cytotoxic. The material showed a two-stage release of curcumin. There was a rapid release for the first 10 days, followed by a slower release up to 30 days [164].

## 5. Development Perspectives

**Figure 10:**
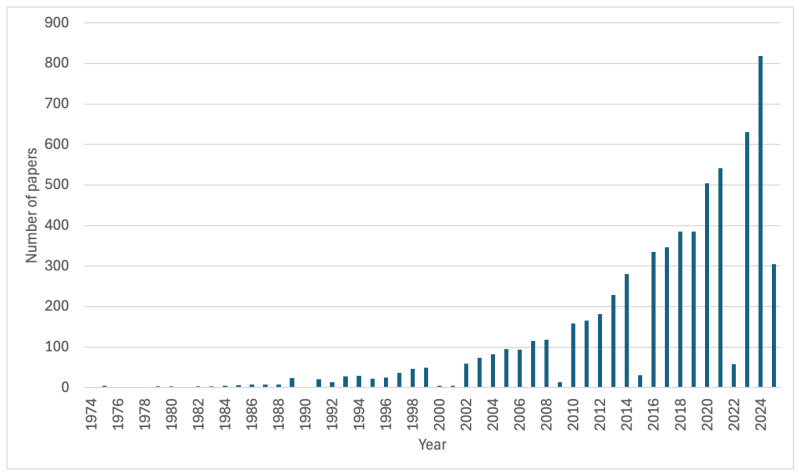
Evolution of publications on biomaterials in dental applications based on PubMed database as of 26 April 2025.

The PubMed database contains over 4000 scientific articles related to biomaterials in dental applications (Figure 10). Publication trends show a dynamic increase in research interest in this field, especially in recent years.

The future of natural polymers in dentistry is strongly oriented toward enhancing their functional properties and expanding their clinical applications. Chitosan, cellulose, collagen, alginate, and hydroxyapatite have already demonstrated significant potential in periodontal regeneration, dental caries prevention, and implantology. Going forward, research is increasingly focused on modifying these biomaterials at the molecular level to improve their mechanical strength, bioactivity, and degradation profiles. Advances in nanotechnology allow the fabrication of nanoparticle-reinforced composites, enhancing drug delivery systems and scaffolds for tissue engineering. In periodontal therapy, the development of bioactive membranes and injectable hydrogels based on chitosan and collagen could lead to more effective regenerative outcomes. Similarly, in caries prevention, polymers like chitosan are being explored for their antimicrobial properties and ability to deliver remineralizing agents directly to affected tissues. For implantology, the combination of hydroxyapatite with bioactive coatings derived from natural polymers aims to improve osseointegration and long-term implant stability. Emerging trends such as 3D bioprinting and biofabrication further expand the possibilities for patient-specific scaffolds and coatings. Although natural polymers present promising biological profiles, overcoming challenges related to their variability, mechanical limitations, and long-term clinical performance will be essential. Overall, the convergence of material science, biotechnology, and nanomedicine is poised to significantly advance the role of natural biomaterials in next-generation dental therapies.

## 6. Conclusions

Natural biomaterials have revolutionized modern dentistry, offering a sustainable and biocompatible alternative to synthetic materials. Their properties—such as biocompatibility, biodegradability, and low immunogenicity—make them highly promising for a wide range of dental applications. From periodontal disease treatment to caries prevention and implantology, these materials play a crucial role in advancing dental care while supporting wound healing and tissue regeneration.

Polymers such as alginate, chitosan, and cellulose have been extensively utilized in the development of advanced dressings and drug delivery systems. Chitosan, with its antibacterial properties and strong adhesion, shows significant potential in combating oral infections and facilitating tissue healing. Cellulose and its derivatives are instrumental in manufacturing bioactive membranes that enhance tissue regeneration. Hydroxyapatite, a biomimetic bone substitute, provides superior biological integration compared to synthetic alternatives, further improving implant success rates. Additionally, collagen has emerged as a key component in periodontal therapy, effectively promoting tissue reconstruction following surgical interventions.

Beyond their therapeutic applications, natural biomaterials are shaping the future of preventive dentistry. Their ability to inhibit plaque formation by suppressing bacterial growth and supporting enamel remineralization presents a natural solution to combat dental caries. Furthermore, the integration of biomaterial-based coatings in dental implants offers not only antibacterial protection but also an accelerated regeneration of soft and hard tissues. The incorporation of plant-derived bioactive compounds into these formulations represents an exciting frontier, potentially replacing synthetic additives with naturally derived alternatives that offer enhanced safety and efficacy.

Despite their immense promise, continued research is essential to optimize the physical and biological characteristics of these biomaterials. Advances in material science, nanotechnology, and bioengineering will enable the refinement of these substances, ensuring their stability, effectiveness, and long-term clinical viability. Future innovations may lead to the development of smart biomaterials with enhanced regenerative capabilities, personalized dental treatments, and bioactive formulations that actively promote oral health.

The ongoing exploration of natural biomaterials holds the key to a new era in dentistry—one that prioritizes biocompatibility, sustainability, and patient well-being. By harnessing the potential of nature-driven solutions, the future of dental therapies will be more effective, safer, and better tolerated, paving the way for groundbreaking advancements in oral healthcare.

## Figures and Tables

**Table 1:** Analysis of the applications of natural biomaterials in dentistry.

Biomaterial	Main Application	Clinical Effectiveness	Limitations	Cost-Effectiveness	Level of Available Evidence
Alginate	Dental impressions, wound dressings, drug delivery, tissue engineering	Good for dental impressions; wound dressings; tissue engineering scaffolds	Poor mechanical properties; needs crosslinking for load-bearing applications	High (low cost, widely available)	High (well-established use in dentistry and wound healing)
Cellulose	Wound dressings, tissue engineering scaffolds, bone repair, cartilage regeneration	Good scaffold material; used for wound dressings and cartilage/bone engineering	Lacks antibacterial properties; needs modification	High (abundant natural sources, cheap bacterial production)	High (widely researched in tissue engineering)
Chitosan	Wound healing, drug delivery, tissue engineering, antimicrobial applications	Antibacterial, antifungal; hemostatic properties; promising for drug delivery	Variable antimicrobial effects depending on molecular weight; solubility limitations	Moderate (cost depends on source and degree of deacetylation)	High (extensive biomedical research, though some variability in results)
Collagen	Tissue scaffolds, joint disease treatments, skin regeneration, hemostatic agents	Excellent biocompatibility; widely used in regenerative medicine and surgery	Risk of immunogenicity if not purified; variable degradation rates	From moderate to high (costly extraction and purification)	Very high (extensive clinical use, FDA-approved products)
Cyclodextrins	Drug delivery systems, pharmaceutical formulations, enhancing drug bioavailability	Effective drug carriers; improve solubility and stability of active compounds	Limited mechanical applications; mainly chemical carriers	Moderate (depends on derivative used)	High (several cyclodextrin-based drugs approved)
Gelatin	Wound healing, drug delivery, tissue regeneration, hemostatic preparations	Good for wound dressings, hemostats, drug delivery; promotes cell adhesion	Poor mechanical properties; stability issues; toxicity risk if chemically crosslinked	High (inexpensive, especially bovine/porcine sources)	High (extensively studied and used clinically)
Hyaluronic Acid	Joint treatments, wound healing, cosmetics, tissue regeneration	Excellent tissue regeneration properties; used in dermatology and joint injections	Rapid degradation; biological effects vary with molecular weight	From moderate to high (depends on molecular weight and source)	Very high (extensively used in orthopedics, dermatology, dentistry)
Hydroxyapatite	Bone repair, dental applications, bone substitutes, drug carriers	Promotes bone regeneration; osteoconductive; widely used in bone implants	Brittle; lacks antimicrobial properties; requires composites for enhanced properties	Moderate (natural and synthetic sources available)	Very high (long history of clinical use in orthopedics and dentistry)
Silk	Drug delivery (cancer therapy), tissue scaffolds, sutures, biomedical engineering	Biocompatible scaffolds; slow degradation; promising for advanced drug delivery	No inherent antibacterial activity; suture biofilm formation	Moderate (depends on production method—traditional or recombinant)	High (growing clinical research; some FDA-cleared products like sutures)

## Data Availability

Data are contained within the presented article.
